# Case Report: Twenty years of metreleptin therapy in congenital generalized lipodystrophy type 1: the longest reported follow-up to date

**DOI:** 10.3389/fendo.2026.1815903

**Published:** 2026-05-13

**Authors:** Elise Van der Borght, Bart Van der Schueren, Roman Vangoitsenhoven, David Cassiman, Baris Akinci, Rebecca J. Brown, Elif A. Oral, Ann Mertens, Pieter-Jan Martens

**Affiliations:** 1 Department of Endocrinology, University Hospitals Leuven – Catholic University (KU) Leuven, Leuven, Belgium; 2 Clinical and Experimental Endocrinology, Department of Chronic Diseases and Metabolism - Catholic University (KU) Leuven, Leuven, Belgium; 3 Department of Gastroenterology-Hepatology and Metabolic Center, University Hospitals Leuven, Leuven, Belgium; 4 Izmir Biomedicine and Genome Center & Dokuz Eylül University Technopark (DEPARK), Dokuz Eylul University Health Campus, Izmir, Türkiye; 5 Devision of Metabolism, Endocrinology and Diabetes, Department of Internal Medicine, University of Michigan Medical School, Ann Arbor, MI, United States

**Keywords:** compliance, AGPAT2, congenital generalized lipodystrophy type 1, metreleptin therapy, pregnancy

## Abstract

Congenital generalized lipodystrophy (CGL) is a rare disorder marked by near-total loss of adipose tissue and severe metabolic disturbances due to leptin deficiency and the inability to store nutrients in adipose tissue effectively. Metreleptin is the only approved leptin replacement therapy for this condition. Here we present the >20-year follow-up of two sisters with CGL type 1 (AGPAT2 deficiency, OMIM# 608594), enrolled in early metreleptin trials. Clinical outcomes, adherence, immunogenicity, and pregnancies were assessed. Both cases showed rapid and sustained metabolic improvement after metreleptin initiation, allowing insulin discontinuation and triglyceride normalization. Menstrual cycles resumed within six months; allowing both to carry successful pregnancies while continuing metreleptin. Both cases experienced reduced treatment adherence over time, linked to psychological distress. One case developed both anti-drug antibodies and neutralizing activity through immune based assay after 14 years, but without significant clinical impact. In conclusion, these cases highlight both the sustained metabolic benefits of therapy and the complex challenges that may arise over time, such as antibody formation and difficulties in maintaining long-term adherence. Documentation of unmet medical needs can provide guidance and impetus for improved therapeutic approaches to achieve optimum quality of life.

## Introduction

Congenital generalized lipodystrophy (CGL) is a rare autosomal recessive disorder, characterized by a selective absence of subcutaneous adipose tissue. Adipose tissue is a metabolically active endocrine organ composed of adipocytes that secrete important adipocytokines. Among these, leptin is a central regulator of energy balance, glucose homeostasis, lipid metabolism, and neuroendocrine and immune function (1). In CGL1, AGPAT2 deficiency restricts the capacity of the adipose tissue to expand and is associated with a (near) absolute deficiency of leptin. This underlies the profound metabolic disturbances, including the development of severe insulin resistance contributing to difficult-to-treat diabetes, polycystic ovary syndrome, severe hypertriglyceridemia and lipid accumulation in ectopic organs such as the liver. Secondary end-organ complications, including steatohepatitis, liver cirrhosis and eventually liver failure, acute pancreatitis, kidney failure and cardiac complications contribute to the high morbidity and mortality observed in individuals with CGL (2). To quantify disease severity in CGL, the Lipodystrophy Severity Score (LDS) provides a standardized assessment of fat distribution, metabolic complications, and key clinical domains including diabetes, cardiovascular, liver, kidney, and reproductive involvement. This tool allows clinicians to monitor disease progression and evaluate the impact of therapeutic interventions (3).

The primary therapeutic approach to treating these metabolic complications is leptin replacement therapy. Recombinant human leptin (metreleptin) mimics the physiological effects of leptin through activation of the leptin receptor and is therefore indicated as an adjunct to dietary management in CGL (4), a use for which it was approved by the U.S. Food and Drug Administration (FDA) in February 2014 and by the European Medicines Agency (EMA) in July 2018 for adults and children aged ≥2 years. This regulatory milestone was supported by two open-label, investigator-sponsored trials (ClinicalTrials.gov identifiers: NCT00005905 and NCT00025883) and their long-term follow-up (5). Here, we present the extended longitudinal follow-up of two of the original trial participants (participants 4 and 5, respectively case 1 and 2), who now represent two of the longest-treated individuals with leptin replacement therapy worldwide (6). Participant 1 unfortunately died and participants 2 and 3 stopped metreleptin early, making these two sisters unique for long-term follow-up and, providing unprecedented insights into the durability, safety, and metabolic impact of chronic metreleptin therapy. The study was approved by the Ethics Committee Research UZ Leuven, and both patients provided written informed consent for participation and publication (S-number S 1196).

## Case series presentation

The two longest-treated individuals with leptin replacement therapy worldwide are two Belgian sisters, aged 39 and 42 years, diagnosed with congenital generalized lipodystrophy (CGL) due to 1-acylglycerol-3-phosphate O-acyltransferase 2 (AGPAT2) deficiency. The initial diagnosis was made at 10 and 12 years of age, prompted by severe metabolic complications, most notably poorly controlled diabetes. The physical features of lack of adipose tissue were present at birth.

At initiation of metreleptin therapy, both individuals were under 18 years of age. They exhibited hypertriglyceridemia alongside poorly controlled diabetes, despite receiving extremely high insulin doses (1200–3000 units/day), consistent with profound insulin resistance. More specifically, case 1 exhibited an initial HbA1c of 7.6%, triglyceride levels of 322 mg/dL and a lipodystrophy severity score (LDS) of 65, while case 2 presented with an HbA1c of 9.5%, triglyceride levels of 913 mg/dL and a LDS of 73. For comparison, in the cohort of females with generalized lipodystrophy described by Brown RJ et al., the median LDS was 46 (3).

Ultrasound examination in both individuals revealed hepatomegaly with increased echogenicity consistent with hepatic steatosis. Due to poor metabolic control using conventional therapy, metreleptin treatment was initiated under the NIH-intramural trial NCT00005905, investigating leptin replacement in individuals with clinically significant lipodystrophy and low circulating leptin levels (<4.0 ng/mL in females), consistent with levels observed in these individuals (0.8 ng/mL and 1.1 ng/mL) (6). As outlined in the study protocol and reported previously, children under the age of 18 were started on a dose of 0.015 or 0.02 mg per kilogram of body weight per day (in males and females respectively) (6). These represented the 50% of estimated physiologic replacement doses. However, the study implemented a dose-escalation approach, starting at 50% of the estimated dose and gradually increasing up to 200%. Following completion of the trial, both patients continued metreleptin therapy under an Expanded Access Program (EAP), enabling long-term follow-up beyond the study period.

**Figure 1:**
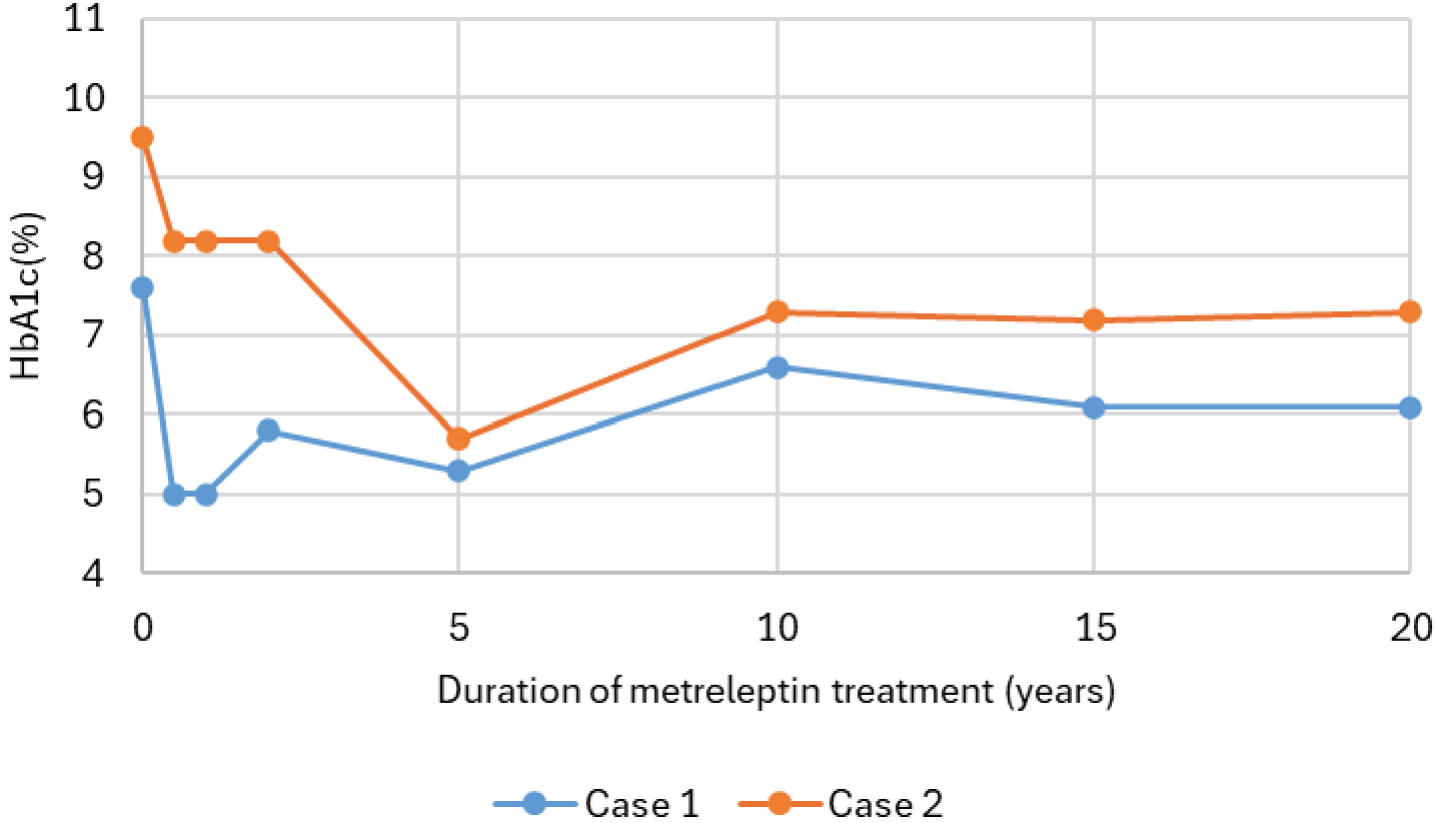
Longitudinal course of HbA1c (%) over the years during metreleptin therapy in two individuals with congenital generalized lipodystrophy type 1 (CGL type 1). HbA1c levels were assessed at baseline, 6 months, 1, 2, 5, 10, 15, and 20 years. In Case 2, metreleptin therapy was temporarily interrupted after 9 months and restarted 5 months later. After a further 7 months, therapy was again interrupted and subsequently restarted. HbA1c, glycated hemoglobin A1c.

**Figure 2:**
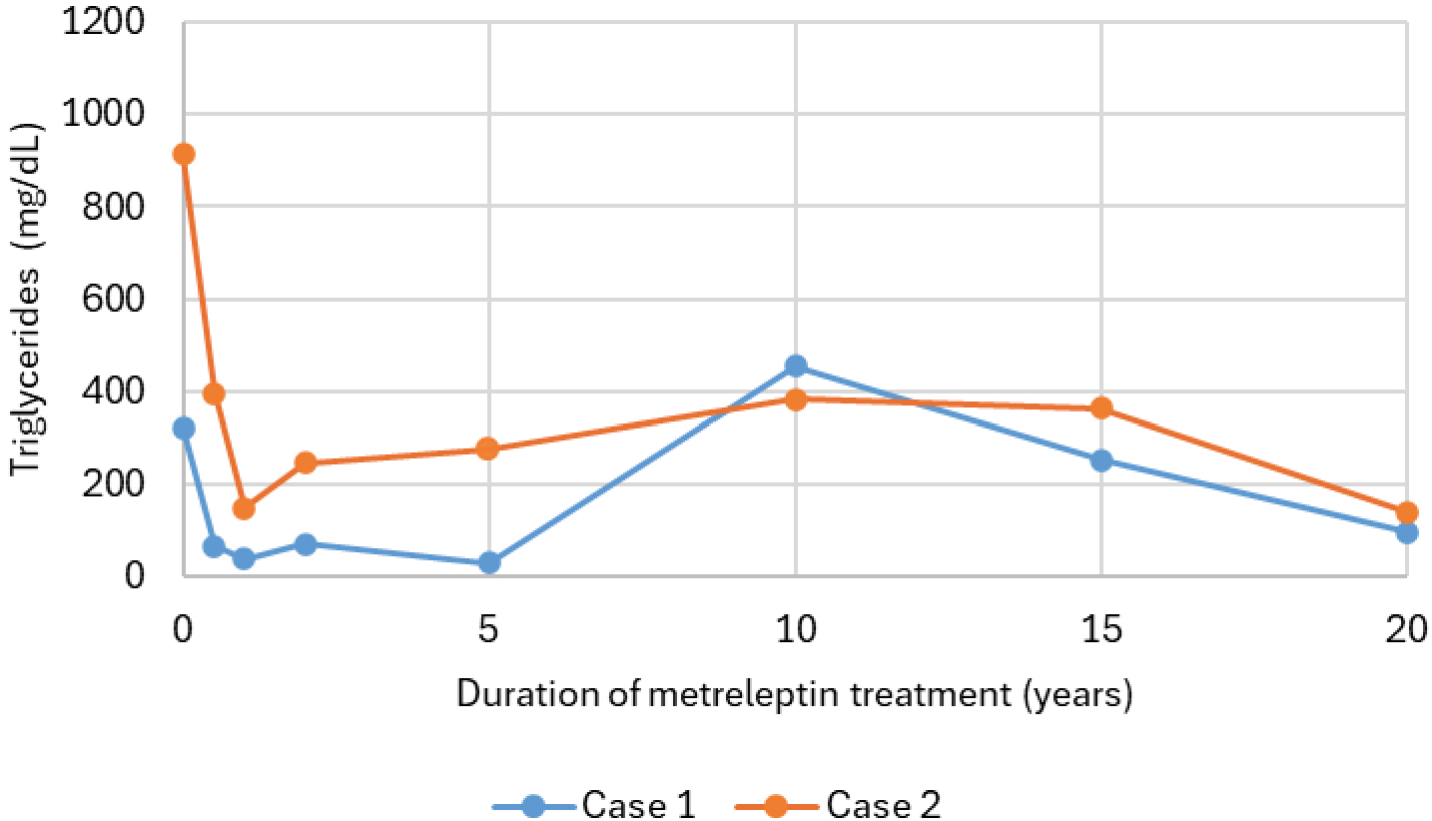
Longitudinal course of triglycerides (mg/dL) over the years during metreleptin therapy in two individuals with congenital generalized lipodystrophy type 1 (CGL type 1). Triglyceride levels were assessed at baseline, 6 months, 1, 2, 5, 10, 15, and 20 years. In Case 2, metreleptin therapy was temporarily interrupted after 9 months and restarted 5 months later. After a further 7 months, therapy was again interrupted and subsequently restarted.

Case 1 responded well to metreleptin therapy, reporting no systemic side effects. In contrast, case 2 experienced a hypertensive episode after the initial injection, necessitating a dose reduction to half of the standard starting dose, with appropriate dose escalation thereafter. Within six months of treatment initiation, both cases showed marked improvement in LDS, decreasing from 65 to 36 in case 1 and from 73 to 46 in case 2, along with marked improvement in hypertriglyceridemia, liver function tests and glycemic control (**Figures 1**, **2**; **Table 1**). Notably, despite requiring very high insulin doses prior to treatment, both were able to discontinue insulin entirely while maintaining stable glycemic control. In case 2, metreleptin therapy was stopped after nine months due to non-adherence and patient volition, requiring temporary insulin use. Metreleptin was restarted five months later, and insulin could be discontinued within five months after reinitiation. However, treatment was interrupted once more due to non-adherence. After the final reinitiation, metreleptin was continued without further interruptions.

**Table 1:** Clinical and laboratory characteristics of the individuals before treatment, after 6 months, and after 20 years of metreleptin therapу.

Metreleptin	Case 1			Case 2			Reference Range
	Before	6 months	20 years	Before	6 months	20 years	
Age (years)	17	18	37	15	16	35	
Height (m)	1.70	1.70	1.70	1.73	1.73	1.73	
Weight (kg)	63.5	54.7	59.2	76.5	71.5	60	
BMI (kg/m²)	21.97	18.83	20.25	25.56	23.89	20.05	
Blood pressure (Systolic/Diastolic, mmHg)	130/90	110/75	161/79	140/80	120/85	94/68	
HbA1c (%)	7.6	5	6.1	9.5	8.2	7.3	(4.0 - 6.0)
Fasting insulin (µU/ml)	211	117	–	115	557	–	
Fasting C-peptide (nmol/L)	0.89	1.42	-	1.89	2.95	1.61	
Oral antidiabetic agents	none	none	metformin	none	none	none	
Total insulin dose (units/day)	1200	0	0	3000	0	14	
Triglycerides (mg/dL)	322	143	96	913	327	141	(<150)
Cholesterol (mg/dL)	259	109	123	232	166	180	(<190)
HDL (mg/dL)	40	30	42	36	36	44	(>45)
LDL (mg/dL)	179	74	80	138	114	107	(<70)
Lipid-lowering drugs	none	none	none	none	none	none	
Creatinine (mg/dL)	0.4	0.4	0.75	0.81	0.7	0.76	(0.51 - 0.95)
Albumin-to-creatinine ratio (mg/g)	1440	17.5	16	1638	196	58	(<30)
ALT (U/L)	138	22	14	75	22	16	(<31)
AST (U/L)	135	13	16	58	22	13	(<31)
ALP (U/L)	172	111	53	89	73	64	(35 - 105)
GGT (U/L)	119	22	26	61	45	28	(<40)
Metreleptin dose (mg/day)	0	5.15	5.65	0	5.15	11.3	
Baseline leptin (ng/mL)	1.1	-	-	0.8	-	-	
Lipodystrophy Severity Score	65	36	41	73	46	49	

BMI, body mass index; Sys, Dia, systolic, diastolic blood pressure; HbA1c, glycated hemoglobin A1c; pmol/L, picomoles per liter; nmol/L, nanomoles per liter; U, units; TG, triglycerides; HDL, high-density lipoprotein; LDL, low-density lipoprotein; mg/dL, milligrams per deciliter; Creatinine, serum creatinine; ALT, alanine aminotransferase; AST, aspartate aminotransferase; ALP, alkaline phosphatase; GGT, gamma-glutamyl transferase; U/L, units per liter; kg, kilograms; m, meters.

## Pregnancy

**Figure 3:**
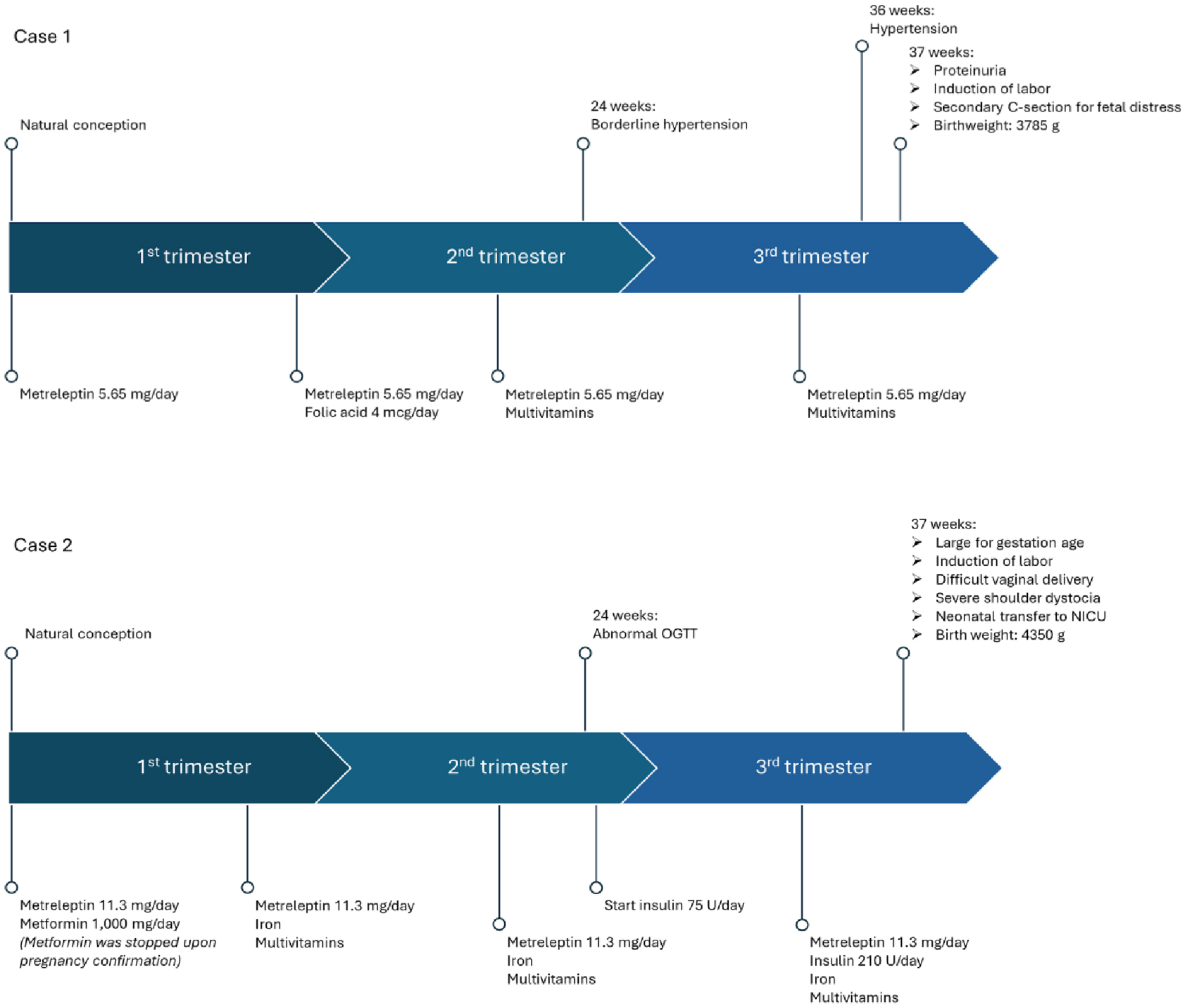
Timeline illustrating the chronological sequence of medical evaluations and treatments during pregnancy for both cases. U, units; mg, milligrams; OGTT, Oral Glucose Tolerance Test; NICU, Neonatal Intensive Care Unit.

**Figure 4:**
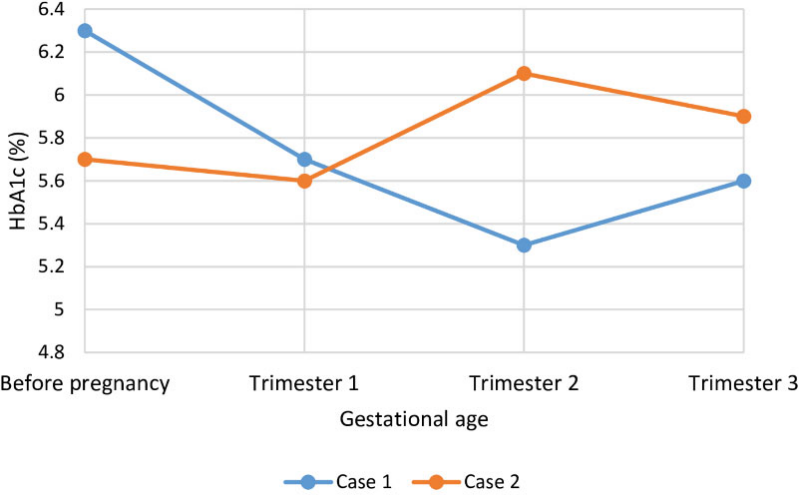
Course of HbA1c (%) during pregnancy while on treatment with metreleptin in two individuals with congenital generalized lipodystrophy type 1 (CGL type 1). HbA1c levels are presented preconception and for each trimester of pregnancy. HbA1c, glycated hemoglobin A1c.

**Figure 5:**
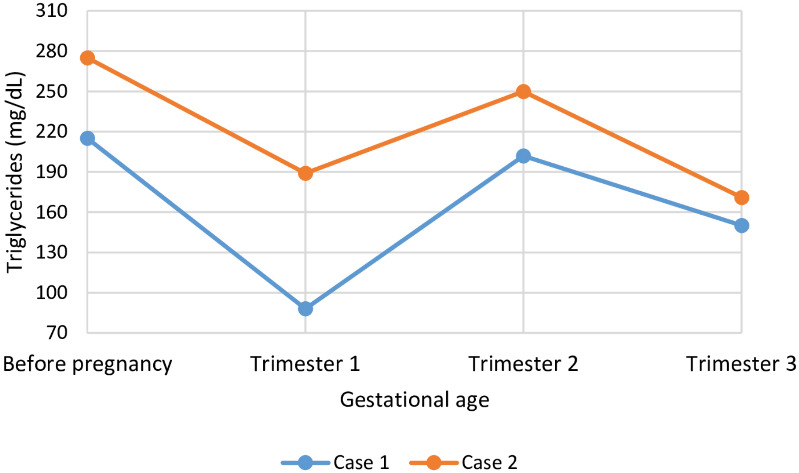
Course of triglycerides (mg/dL) during pregnancy while on treatment with metreleptin in two individuals with congenital generalized lipodystrophy type 1 (CGL type 1). Triglyceride levels are presented preconception and for each trimester of pregnancy.

At the time of metreleptin initiation, both individuals were adolescents (aged 15 and 17 years) and had not yet experienced menarche. Within six months of starting therapy, menstrual cycles began in both cases. In case 1, this led to an unplanned pregnancy at age 22, which was electively terminated. Later, case 2 became pregnant while still enrolled in the clinical trial, despite the requirement to use effective contraception and the lack of safety data for metreleptin during pregnancy. Although pregnancy is explicitly listed as a contraindication in the SmPC and metreleptin is not recommended for use in pregnant patients, given her strong desire to conceive and the absence of effective alternatives to manage CGL1 during pregnancy, a decision was made to continue metreleptin throughout the pregnancy. Case 1 subsequently conceived in a planned fashion at the age of 33 years, after 16 years of metreleptin therapy following the approval in Europe (**Figures 3**–**5**).

At the onset of pregnancy, case 1 was treated with metreleptin monotherapy, without the use of insulin, oral antidiabetic agents, or lipid-lowering medications. Case 2, however, was taking 1, 000 mg of metformin at the time of conception in addition to metreleptin therapy (**Figure 3**). Metformin was stopped upon pregnancy confirmation. Metreleptin was continued at the preconception dose, and both cases remained metabolically stable throughout pregnancy. During the first half of gestation, glycemic targets were achieved without insulin therapy in either case. However, in case 2, an abnormal oral glucose tolerance test at 24 weeks’ gestation prompted the initiation of insulin therapy at a dose of 75 units per day. This dose was gradually increased to 210 units daily in the third trimester to maintain glycemic control. Case 1 did not require insulin at any point during pregnancy. Lipid profiles remained excellent in both cases, and no adjustment to the metreleptin dose was necessary.

Up to 36 weeks of gestation, both pregnancies progressed without complications. At 36 weeks, case 1 was admitted to the maternal intensive care unit due to hypertension. At 37 weeks, new-onset proteinuria raised suspicion of preeclampsia, prompting induction of labor. A secondary caesarean section was performed due to fetal indications. A male neonate was delivered at 37 weeks with a birth weight of 3, 785 grams. He was in good clinical condition, with no neonatal complications, congenital anomalies, or other health problems. Currently, he remains in good health aside from attention-deficit/hyperactivity disorder (ADHD).

In case 2, labor was induced at 37 weeks due to suspected large-for-gestational-age (LGA) fetus. Delivery was complicated by obstructed labor, which may have been influenced by altered pelvic soft tissue or cephalopelvic disproportion, as previously reported for this patient (7). Despite this, vaginal delivery was achieved. The male neonate, with a birth weight of 4, 350 grams (macrosomia), experienced shoulder dystocia, resulting in Erb’s palsy. His postnatal course was complicated, including cardiac arrest requiring immediate resuscitation, pneumothorax requiring chest tube placement, a second cardiac arrest, and three weeks of ventilatory support with EEG monitoring. He was discharged after 1.5 months. Currently, he has residual weakness of the left arm without functional impairment, noticeable only during physical exercise. Otherwise, he is in good health, with normal pubertal development and no learning disorders.

Both mothers reported breastfeeding while continuing metreleptin therapy. Case 2 breastfed for six months. Case 1 also breastfed, although the exact duration was not documented. No adverse effects related to metreleptin exposure were reported in either infant during the lactation period.

## Long-term outcomes

**Figure 6:**
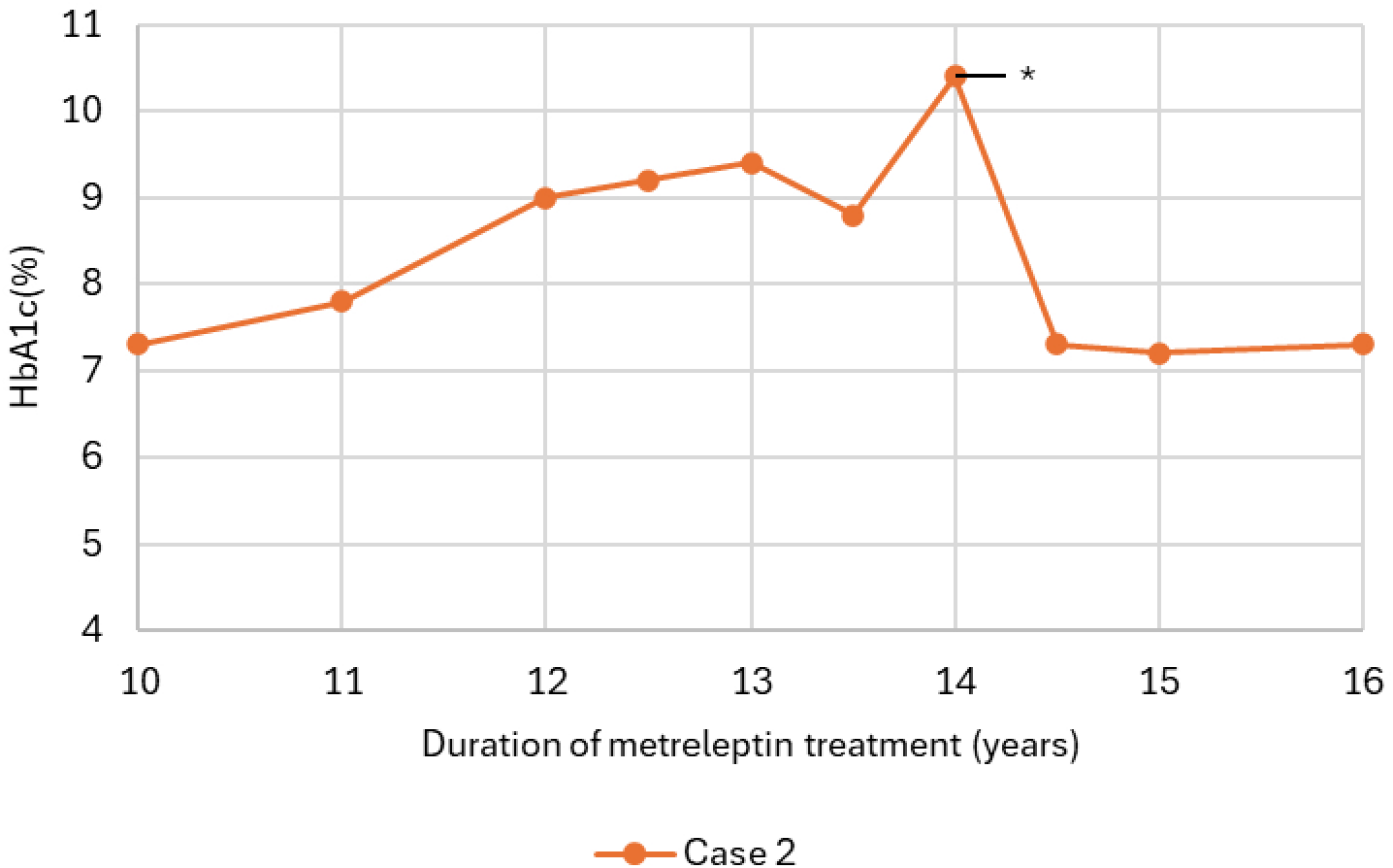
HbA1c trajectory of case 2, 10–16 years after initiation of metreleptin therapy, showing deterioration prior to the asterisked time points (*), when anti-drug antibodies (ADA) to leptin were assessed. Following optimization of the injection regimen and improved adherence, HbA1c decreased despite an unchanged total daily metreleptin dose, arguing against a clinically meaningful impact of ADA to leptin on metabolic control. *After 14 years of therapy, antibodies were discovered.

In case 1, long-term metabolic control was sustained for over 20 years under metreleptin therapy, with only occasional periods of suboptimal control attributable to incomplete treatment adherence (**Figures 1**–**6**, **Table 1**). This lasting metabolic improvement was accompanied by a significant reduction in LDS. The LDS initially decreased from 65 at baseline to 36 after six months. While a slight increase to 41 was noted after 20 years, the LDS remained substantially lower than baseline. Adherence to metreleptin therapy was occasionally challenged by the burden of daily injections and local injection-site discomfort. In addition to these treatment-related challenges, she also experienced recurrent musculoskeletal complaints and psychological distress, both of which likely contributed to fluctuating adherence.

In case 2, metabolic control diminished after 14 years under metreleptin therapy as suggested by increased fasted glucose and insulin levels, with the need to initiate long-acting insulin therapy, together with recurrence of hypertriglyceridemia, albeit both relatively well controlled compared to levels prior to initiation of metreleptin (**Figures 1**, **2**, **6**; **Table 1**). This aligns with the LDS, demonstrating a pronounced initial improvement at six months, with a decrease from 73 at baseline to 46 at six months, followed by a slight increase to 49 after 20 years, still reflecting a substantial long-term improvement compared to baseline. The decline in treatment success, despite many years of reasonable control with the same dose of metreleptin, led to suspicion for the development of anti-drug antibodies (ADA) to leptin, potentially leading to neutralizing activity and reduced *in vivo* efficacy of metreleptin (**Figure 6**). Antibodies were assessed using a validated electrochemiluminescence immunoassay (ECLIA) binding assay to detect ADA, with samples confirmed positive subsequently tested in a receptor-blocking assay to evaluate neutralizing activity with titers determined by serial dilution, expressed as the percentage inhibition of metreleptin binding. ADA were present (titer of 1:1250), with neutralizing activity to leptin being detected (titer of 1:30, inhibition level of 58.54% at the minimal required dilution) (8). As the clinical implications of developing ADA with neutralizing activity, as well as the clinically relevant titer and percentage-inhibition thresholds are not well characterized, metreleptin therapy was continued with injections divided over two sites instead of one to improve absorption (9). In addition, case 2 experienced emotional challenges, likely contributing to suboptimal adherence. After implementing the modified injection regimen and achieving better adherence, metabolic control improved despite continuation of the same total daily metreleptin dose. She remained stable thereafter despite antibodies persisting at comparable titers (even slightly higher titers were measured due to improvement of assay sensitivity). Based on these findings, neither ADA nor neutralizing antibodies were considered responsible for the observed metabolic deterioration.

## Discussion

Here we present, to our knowledge, the longest follow-up to date of more than 20 years on leptin-replacement therapy, under the form of a recombinant analogue of human leptin, namely metreleptin. Metreleptin was approved by the FDA and EMA only in 2014 and 2016 respectively, but here we present two individuals living with CGL, who have been treated with metreleptin from the earliest investigator-sponsored trials. The individuals presented here have CGL type 1, caused by AGPAT2 deficiency. The mechanisms by which AGPAT2 deficiency leads to lipodystrophy remain incompletely understood, but may involve impaired lipogenesis, altered differentiation of preadipocytes to adipocytes, defective adipogenesis, increased adipocyte loss through apoptosis or necrosis, and inflammation triggered by accumulation of lysophosphatidic acid (LPA) in adipose tissue and liver (10, 11). Individuals affected by CGL have low levels of leptin, which, along with inability to store nutrients in adipose tissue, leads to profound metabolic disturbances. Consistent with this, the two individuals in this report presented with severe hypertriglyceridemia and severe insulin resistance, leading to difficult-to-treat diabetes, as shown by an extraordinarily increased insulin demand relative to common forms of diabetes, all from an extremely young age.

Initiation of metreleptin therapy in both cases resulted in significant improvements in metabolic control, with notable reductions in HbA1c and triglyceride levels within the first six months of treatment. These outcomes are consistent with previous studies, further corroborating their findings and extending them by demonstrating durable metabolic benefits in the longest follow-up to date (5, 12, 13). Consistent with earlier observations (3), LDS decreased sharply soon after the start of metreleptin therapy, reflecting a reduction in overall disease burden. A slight rise in LDS was noted in the following years. However, this pattern likely reflects the interplay of factors, including age-related changes in organ function, cumulative metabolic stress, and gradual progression of disease-related complication, rather than by loss of therapeutic efficacy. In case 2, however, a decline in metabolic control was noted after approximately 14 years of metreleptin therapy. When metabolic deterioration occurs during treatment with a therapeutic protein, the development of both ADA and neutralizing activity to leptin should be considered as contributor. The formation of antibodies against therapeutic proteins is a well-recognized phenomenon, and metreleptin, as a recombinant analogue of human leptin differing by an additional N-terminal methionine, is no exception. Antibody formation against metreleptin is reported to be very frequent (detected in >85-90% of cases) and may lead to a decrease in metabolic efficacy of the therapy in very select cases (8, 14). The underlying mechanisms of antibody formation are not yet fully elucidated, but may be related to the physicochemical properties of metreleptin and the non-physiological route of administration in those lacking subcutaneous adipose tissue, resulting in direct injection into the circulation (14). Unfortunately, no functional assays are currently available to reliably assess the impact of antibody formation on circulating leptin levels, partly because antibody binding itself can interfere with these measurements. As the level of antibody formation in our case was considered to be mild, the administration of metreleptin was optimized by using short, fine needles in the abdominal region, limiting injection volume with the option to divide it between two sites, and applying manual pressure to reduce discomfort improved tolerability and support adherence, as already suggested by others (13).

While the daily requirement of subcutaneous injections likely contributed to suboptimal adherence, in both cases, psychosocial factors also played a significant role. This underscores the importance of incorporating mental health support into the long-term management of chronic conditions, an aspect of care that has only recently begun to receive more attention in the literature.

To improve long-term treatment adherence, alternatives such as mibavademab (REGN4461), a monoclonal leptin receptor agonist administered once weekly, may offer a less burdensome treatment option. Based on promising phase 1 data showing improvements in triglycerides and hepatic steatosis in a compassionate-use case (15), mibavademab was recently evaluated in a randomized, double-blind, placebo-controlled phase 2 trial (NCT04159415) in individuals with congenital or acquired generalized lipodystrophy. Preliminary findings indicate that the drug was well tolerated; however, after 8 weeks on low-dose mibavademab clinically meaningful changes in HbA1c or fasting triglycerides were not observed compared with placebo (16, 17). Long-term outcomes (the protocol envisages up to 128 weeks) are currently being assessed (16). Further studies are warranted to confirm efficacy and better define the therapeutic role of mibavademab in lipodystrophy management.

The two cases reported here also provide insight into the management of pregnancy in women with CGL treated with metreleptin. In both cases, metreleptin restored menstrual cycles, facilitating conception, and was continued throughout pregnancy at the same dose used prior to conception. Although metreleptin is classified as FDA pregnancy category C, based on animal studies that suggest potential fetal harm, well-controlled human data are lacking and its teratogenic risk therefore remains uncertain (18). From a European regulatory perspective, the EU SmPC for metreleptin states that the drug is not recommended during pregnancy or in women of childbearing potential not using contraception, although no causal relationship with reported adverse pregnancy outcomes has been established. In our case series, continuing metreleptin therapy appeared to confer greater benefits than potential risks, primarily by supporting metabolic stability during pregnancy. Case 1 maintained adequate glycemic control without the need for insulin, whereas case 2 developed gestational diabetes requiring insulin from 24 weeks of gestation. Despite this, metabolic control in case 2 remained relatively stable, and both pregnancies progressed without major metabolic deterioration. These observations in the two cases presented suggest that metreleptin was associated with both the restoration of fertility and maintenance of metabolic stability during pregnancy. However, these findings are specific to these individual patients and may not be generalizable to the wider CGL population. No teratogenic effects were observed, suggesting that continued therapy appears to be well tolerated in this context. Despite adequate metabolic control, careful perinatal monitoring with consideration of caesarean delivery are recommended in similar cases, given the underlying anatomical challenges related to childbirth in individuals with CGL (7).

In summary, these two cases provide valuable long-term insights on metreleptin therapy in CGL. They highlight the sustained metabolic benefits, the potential of antibody formation, challenges in maintaining treatment adherence over time, and key considerations for pregnancy management. A comprehensive, multidisciplinary approach remains essential to optimize outcomes in this rare and complex condition.

## Data Availability

The original contributions presented in the study are included in the article/supplementary material. Further inquiries can be directed to the corresponding author.
